# Lymphoplasmacytic Lymphoma/Waldenstrom Macroglobulinemia Masquerading as IgM Warm Antibody Autoimmune Hemolytic Anemia in Association With Mycoplasma pneumoniae Infection: A Case Report

**DOI:** 10.7759/cureus.31693

**Published:** 2022-11-20

**Authors:** Mohammad Abu-Abaa, Sindhu Chadalawada, Salman Kananeh, Ahmed Hassan, Omar Jumaah

**Affiliations:** 1 Internal Medicine, Capital Health Regional Medical Center, Trenton, USA

**Keywords:** cold agglutinin, mycoplasma pneumonia, autoimmune hemolytic anemia (aiha), lymphoplasmacytic lymphoma, waldenstrom macroglobinaemia

## Abstract

Warm antibody autoimmune hemolytic anemia (AIHA) is mostly of IgG subtype. IgM subtype is extremely rare and has not been reported in association with lymphoplasmacytic lymphoma (LPL)/Waldenström macroglobulinemia (WM). We are reporting the case of a 75-year-old female patient who presented with severe hemolytic anemia and *Mycoplasma pneumoniae* pneumonia (MPP). Cold agglutinin and serum protein electrophoresis (SPEP) were negative but immunofixation was positive for IgM. Ultimately, hemolytic anemia was labeled warm antibody AIHA in association with MPP. She presented again one year later with more severe hemolytic anemia. Persistently elevated IgM was seen in immunofixation and triggered bone marrow biopsy that confirmed LPL/WM. This case highlights the clinical pearl that warm antibody AIHA in association with MPP is a rare entity and more intensive investigation to rule out other etiologies is mandated. Also, this case is rare as it is of IgM subtype warm AIHA and observed in the context of LPL/WM.

## Introduction

*Mycoplasma pneumoniae* infection is a benign self-limiting condition that usually affects those between 5-20 years of age. It can present with pulmonary and extrapulmonary manifestations, the latter is seen in 25% of cases [1]. The most common hematological manifestation is autoimmune hemolytic anemia of two types; the most common is cold agglutinin and rarely warm antibody autoimmune hemolytic anemia (AIHA). Cold agglutinins are IgM antibodies that bind to RBC, which, upon exposure to cold temperature, induce complement fixation and hemolysis [2]. Waldenström macroglobulinemia (WM) is a low-grade B-cell lymphoproliferative disease characterized by small lymphocytes and IgM monoclonal gammopathy. Anemia in WM is usually multifactorial and rarely secondary to AIHA, which is usually of cold agglutinin type. Some patients with primary cold agglutinin disease can eventually transform into full-blown WM [3]. Warm antibody hemolytic anemia in association with WM is rarely reported [4].

## Case presentation

A 75-year-old female patient presented to the emergency department (ED) with a new-onset generalized weakness. At home and two days prior to admission, she fell onto the floor and was not able to stand up. Past medical history is significant for chronic kidney disease, hypertension, dyslipidemia, and chronic obstructive lung disease (COPD). She did not report any respiratory symptoms. Chest x-ray was unremarkable. In the ED, vital signs were stable except for asymptomatic elevated blood pressure at 200/100 mmHg. Physical exam was negative for bleeding, skin rash, acrocyanosis, and skin discoloration. Basic lab work showed normochromic normocytic anemia with hemoglobin level at 10 g/dL and mean corpuscular volume (MCV) of 87 fL. Total bilirubin was elevated at 8.5 mg/dL with direct globin elevated at 3.9 mg/dL. Alkaline phosphatase (ALP) was elevated at 135 (reference range 26-126), aspartate aminotransferase (AST) was elevated at 136 U/L (reference range 14-36), and alanine transaminase (ALT) was high normal at 31 U/L (reference range 14-36 ). In addition, rhabdomyolysis was also evident by elevated serum creatine kinase (CK) level at 16,289 units/L. Viral hepatitis serology was unremarkable. The patient was commenced on IV fluid therapy.

**Figure 1:**
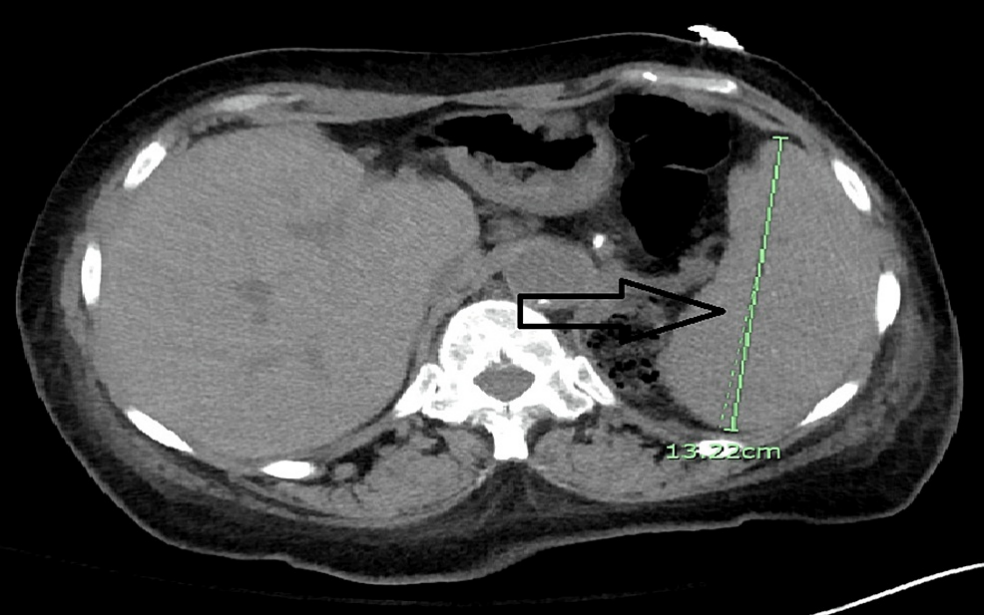
Computed tomography scan of the abdomen showing enlarged spleen.

**Figure 2:**
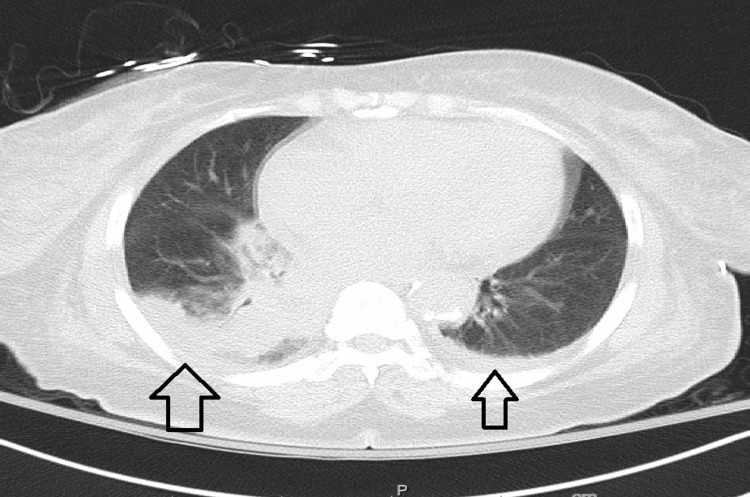
Computed tomography of chest showing bilateral posters-basal infiltration with effusion (arrows).

Within one day of admission, hemoglobin level dropped to 5.2 g/dL. Hemolysis was evident with low haptoglobin less than 10 mg/dL (reference range 46-346). Blood smear was significant for microspherocytes with some agglutination of RBCs and no evidence of schistocytes. Rouleaux phenomena was reported as slight. Lactate dehydrogenase (LDH) was elevated at 698 units/L (reference 122 to 246 units/L). Also, total bilirubin was increased to 6 mg/dl with a decreased fraction of direct bilirubin to 3 mg/dl. Computed tomography (CT) scan of the abdomen and pelvis was remarkable for splenomegaly at 13.2 cm (Figure 1). The clinical examination did not show evidence of lymphadenopathy. Antinuclear antibody (ANA) and other vasculitis workups were unremarkable. CT scan of the chest showed bilateral basal consolidation with effusion (Figure 2). Analysis for *Mycoplasma pneumonia* showed elevated IgG antibody at 222 units/L (reference range 0-100) and elevated IgM antibody at 1,837 (reference range 0-767). Interestingly, analysis for Epstein-Barr virus (EPV) infection also showed positive virus capsid antigen (VCA) IgM antibody, VCA IgG antibody, and Epstein-Barr nuclear antigen (EBNA) IgG antibody. Direct Coombs test was positive for C3 but negative for IgG. The direct agglutination test was positive. However, cold agglutinin was unremarkable. No hemoglobinuria was seen. Immunoglobulin analysis showed elevated IgM with normal levels of other immunoglobulins. Immunofixation showed IgM monoclonal protein with kappa specificity. Serum and urine protein electrophoresis failed to show M spike. The patient received blood transfusion and folic acid with improvement in hemoglobin level. It was presumed warm antibody AIHA, secondary to acute *Mycoplasma pneumoniae* pneumonia, and the patient was treated with aztreonam and tigecycline for seven days. The patient was discharged and lost for follow-up.

**Figure 3:**
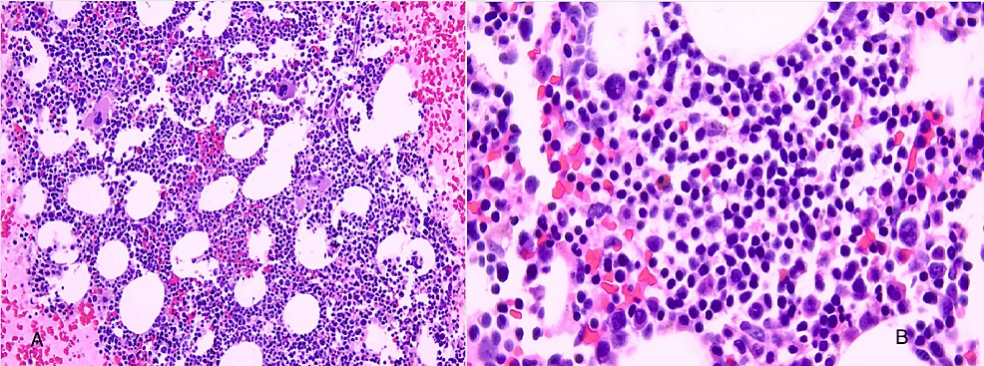
Bone marrow (BM) biopsy showing prominent lymphocytic infiltration with polyclonal plasma cells that constituted at least 50% of BM cellularity.

**Figure 4:**
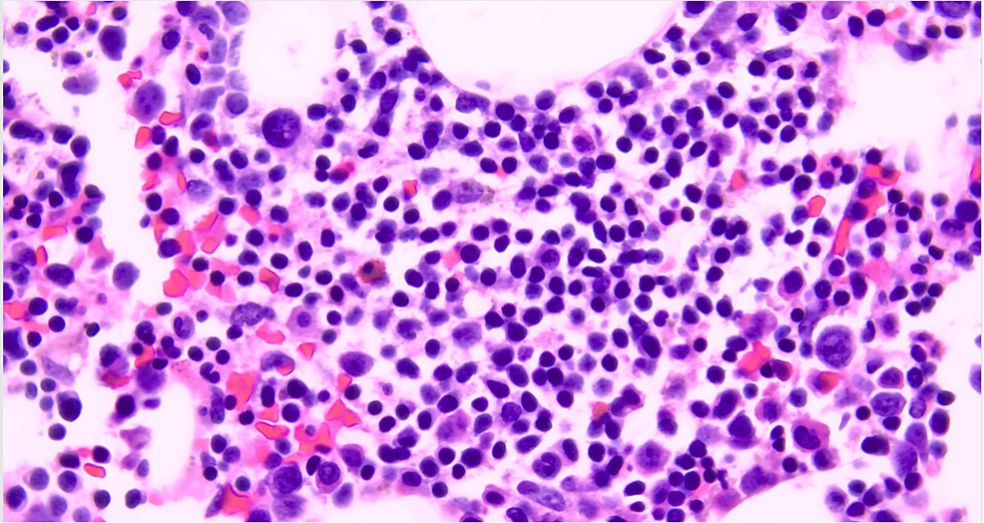
A closer image of bone marrow biopsy showing prominent lymphocytic infiltration.

**Figure 5:**
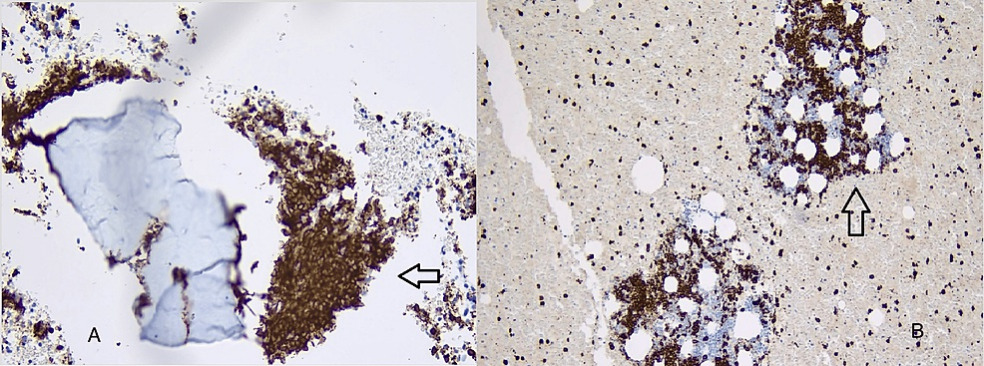
CD-138 staining showing diffuse lymphoplasmacytic infiltrate.

The patient presented again one year later with generalized severe weakness. In the ED, she was found vitally stable with severe normochromic normocytic anemia with hemoglobin level of 3.2 g/dL and MCV of 97 fL. Thrombocytopenia was also evident at 137 cells/ml. Peripheral blood film also showed evidence of microspherocytes, Rouleaux phenomena, and a few schistocytes. Hemolysis was supported by elevated reticulocyte count at 5.3% (reference 0.5-1.8%), elevated LDH at 329 U/L, elevated total bilirubin at 9 mg/dl, direct bilirubin at 3 mg/dl, and reduced haptoglobin less than 18 mg/dl. Serum folate and vita B12 levels were both normal. Coomb’s test was again positive for C3 only and not for IgG. As was seen previously, the direct agglutination test was positive. But, cold agglutinin was tested twice and was persistently negative. The patient did lack features of cold-associated hemolysis and hemoglobinuria. Thus, the type of hemolysis was concluded as warm AIHA. A repeat CT scan of the abdomen pelvis showed unchanged splenomegaly in size. The CT chest was unremarkable. Serum protein electrophoresis was also still negative but urine protein electrophoresis showed a band in gamma. Immunofixation repeat showed persistent similar findings. The kappa light chain was elevated at 1.9 mg/L(reference ranges 3-19) with an elevated kappa lambda ratio of 2.5 (reference is 0.2-1.6). Immunoglobulin analysis showed persistent elevation of IgM with normal other hemoglobin. Bone marrow aspiration flow cytometry showed 11% kappa with B cells with reduced erythroid and myeloid cells. Bone marrow biopsy showed prominent lymphocytic infiltration, the majority of which are clonal kappa-positive B cells (CD20+ and Pax5+) with no specific phenotype (CD5-, CD10-, and CD103-) with polyclonal plasma cells that constituted at least 50% of bone marrow cellularity. This is suggestive of lymphoplasmacytic lymphoma/WM (Figures 3, 4, 5 ).

A tabular format of lab results in both admissions is shown in Table 1. The patient received blood transfusion along with corticosteroids, which improved the degree of hemolysis as well as thrombocytopenia. A dose of rituximab was also given. Despite outlining the care plan for the patient, she was lost for follow-up after discharge.

**Table 1:** Laboratory test findings in both admissions.

Variable	Initial Presentation	1 year later finding	Reference range
White blood cell count	9000	4000	4000-10000 cell/ml
Hemoglobin	4.4	3.2	11-15 g/dl
Platelet	193	134	150-400 cell/ml
Retic%	4.6	5.7	0.5-1.8%
Folate	-	7.3	More than 2.7 ng/ml
Vitamin B12	-	794	240-930 pg/ml
Hepatitis B S Ag	Negative	Negative	Negative
Hepatitis C Ab	Negative	Negative	Negative
HIV	Negative	Negative	Negative
Anti-C3 Coomb	Positive	Positive	Negative
Anti-IgG Coomb	Negative	Negative	Negative
Haptoglobin	Less than 10	Less than 10	40-340 mg/dl
IgA	102	112	60-420 g/dl
IgG	1039	955	580-1600 g/dl
IgM	973	1332	26-217 g/dl
Alpha 1 globulin	0.4	0.3	0-0.4 g/L
Alpha 2 globulin	0.6	0.4	0.4-1 g/L
Albumin	2.5	2.9	2.9-4.4 g/L
Gamma globulin	1.5	1.7	0.4-1.8 g/dl

## Discussion

Hemolytic anemia secondary to *Mycoplasma pneumoniae *infection often occurs during two to three weeks of illness [5]. It can present before, during, or after pulmonary manifestations. It is usually associated with cold agglutinin. Cold agglutinin is seen in 50-60% of cases and is more specific for I antigen on the RBC membrane and usually results in mild, subclinical hemolytic anemia [6]. More severe anemia can be seen in severe pulmonary infection [7] Cold agglutinin is usually of IgM type in 90% of cases [8]. Diagnosis of cold agglutinin hemolytic anemia is based on positive cold agglutinin and positive direct Coomb’s test. Warm antibody hemolytic anemia secondary to *Mycoplasma *infection is rare. Although it can range from mild to severe, It is usually severe and requires transfusion therapy. It can be seen with positive Coomb’s for IgG with or without C3d. Hence, testing for cold agglutinin is required [9]. Warm antibody AIHA positive only for C3 is rare, only seen in 6-13% of cases [10]. 

The best initial step in the evaluation of hemolysis is Coomb’s test. If it is reactive for C3 with/without IgG, the next step is direct agglutination test (DAggT). In our patient, positive DAggT but with persistently low cold agglutinin titer of less than 64, along with lack of features of paroxysmal cold hemoglobinuria including cold associated hemolysis, atypical serological features, and hemoglobinuria pointed toward warm AIHA [11]. The fact that the warm antibody was of IgM type is considered extremely rare [12]. It has been reported in association with Sjögren syndrome, immune thrombocytopenic purpura, severe combined immunodeficiency, eosinophilic granulomatosis, and polyangiitis. No association with WM has been reported to the best of our knowledge. In contrast to IgG-mediated warm AIHA, IgM-mediated warm AIHA has no currently established treatment. Steroids, rituximab, and plasmapheresis have been reported to have limited efficacy [12]. 

Initially in this patient, warm antibody AIHA in the clinical context of pulmonary infection and positive serology for *Mycoplasma* infection pointed toward *Mycoplasma*-associated-warm AIHA. Although the possibility of multiple myeloma and lymphoproliferative disorder was kept in mind and investigated by serum and urine protein electrophoresis and immunofixation, these were largely inconclusive. Splenomegaly was attributed to extravascular hemolysis of warm antibody AIHA and positive immunofixation was attributed to acute infection. However, *Mycoplasma*-associated warm AIHA is an extremely rare entity, and the lesson learned from this case is that the rarity of this condition mandates in-depth investigations to rule out other etiologies. Ultimately, persistent severe hemolysis hinted toward another etiology. 

EBV is a lymphotropic virus and is etiologically associated with multiple lymphomas [9]. It can infect all B cell lines but establishes long-term persistence only in memory B cells. Three main types of B cell lymphomas are associated with EBV including Burkitt lymphoma, Hodgkin, and diffuse large B cell lymphomas. The median interval between the onset of chronic EBV infection and the onset of malignant lymphoma is usually over 10 years (median 37 years) [13]. In the present case, EBV serology can indicate either recovery of acute infection, reactivation, or chronic active infection [14]. Unfortunately, no further EBV serology testing could be done. No clear evidence is found in the English literature to suggest a relationship between EBV and WM. WM has been classically associated with hepatitis C virus and hepatitis serology was nonreactive in our case. 

## Conclusions

AIHA in association with *Mycoplasma* infection is usually mild and subclinical. It is usually of cold agglutinin type. Warm antibody AIHA can occur rarely in association with *Mycoplasma* infection. However, the rarity of such findings, especially If severe hemolytic anemia and with no severe pulmonary manifestation, should raise suspicion of another etiology. Although hemolytic anemia in association with WM is most commonly of cold agglutinin type, warm antibody AIHA can also be seen in rare cases, as in the present one. In both cases, warm antibody AIHA is usually of IgG type. However, this case demonstrates the rarity of IgM-type warm antibody AIHA. 
